# Psychological Wellbeing in Physical Education and School Sports: A Systematic Review

**DOI:** 10.3390/ijerph18030864

**Published:** 2021-01-20

**Authors:** Javier Piñeiro-Cossio, Antonio Fernández-Martínez, Alberto Nuviala, Raquel Pérez-Ordás

**Affiliations:** 1 Department of Sports and Computer Science, Pablo de Olavide University, 41013 Seville, Spain; jpineiro@psicosport.cl (J.P.-C.); anuvnuv@upo.es (A.N.); 2 Faculty of Human Sciences and Education, University of Zaragoza, 22003 Huesca, Spain; rpordas@unizar.es

**Keywords:** mental health, physical activity, educational interventions, emotional education, soft skills

## Abstract

Mental health in children and adolescents has become an increasingly important topic in recent years. It is against this backdrop that physical education and school sports play an important role in promoting psychological wellbeing. The aim of this review was to analyse interventions for improving psychological wellbeing in this area. To this end, a literature review was conducted using four databases (WOS, SPORTDiscus, SCOPUS and ERIC) and the following keywords: psychological wellbeing, physical education, and school sports. Twenty-one articles met the inclusion criteria. The results showed that interventions varied greatly in terms of duration and used a wide range of strategies (conventional and non-conventional sports, physical activity, games, etc.) for promoting psychological wellbeing, primarily among secondary school students. There was a lack of consensus as to the conceptualisation of the construct of psychological wellbeing, resulting in a variety of tools and methods for assessing it. Some studies also suggested a link between psychological wellbeing and other variables, such as basic psychological needs and self-determination. Finally, this study provides a definition of psychological wellbeing through physical activity based on our findings.

## 1. Introduction

Mental health problems in adulthood originate primarily in childhood and can be related to a variety of causes, such as socioeconomic, genetic or cultural factors [1]. It is in adolescence that the greatest risks of behaviours affecting wellbeing occur [2,3]. The World Health Organisation (WHO) [4] describes these problems as the main cause of disability worldwide, with depression extremely prevalent among young people [5]. School is therefore a suitable location for interventions, allowing students to acquire and develop skills and strategies to face life’s challenges as best they can, with high levels of physical and psychological wellbeing [6,7].

In recent years, the use of physical activity (PA) and sports for personal and social development in children and adolescents has been the subject of an increasing number of studies. Research suggests that physical education (PE) and school sports provide a suitable, effective framework for transferring and teaching skills and strategies to reduce risky behaviour and promote wellbeing [8,9]. Although there is evidence that PA and sports enhance young people’s skills and values at these life stages, there is a gap in research on their impact on psychological wellbeing and a lack of consensus as to the definition of psychological wellbeing in this context [10].

Studies have shown that individuals with high levels of psychological wellbeing are more successful in terms of education, work, friends, stable relationships and physical health [11]. In education, psychological wellbeing leads to improved attention, creative thinking and holistic thinking [5]. Psychological wellbeing is usually understood as a construct from the eudaimonic tradition. Unlike subjective wellbeing, which derives from happiness and satisfaction through the pursuit of pleasure and the reduction of pain, psychological wellbeing seeks to allow people to attain their maximum potential by developing virtues [12], focusing on capabilities and personal growth, and understanding that happiness is achieved through individual self-realisation [13,14]. Psychological wellbeing focuses on the process and on pursuing values leading to personal growth rather than on pleasurable, pain-avoiding activities, thus making the individual feel alive and authentic [15]. Ryff [16] proposed a multidimensional model for understanding psychological wellbeing called the Integrated Model of Personal Development (IMPD), consisting of six dimensions: self-acceptance, autonomy, personal growth, purpose in life, environmental mastery and positive relations with others.

Although information on interventions in the context of PE and school sports using the IMPD is limited, the model is widely recognised as a coherent, logical, valid construct [12], and PA and sports represent useful tools for its implementation [17]. Two systematic reviews by Malm, Jacobsson and Nicholson [18] and Mnich, Weyland, Jekauc and Schipperijn [19] list the benefits of PA and sports, including, on a physical level, reduced risk of developing metabolic syndromes, reduced side effects of cancer, improved cardiovascular health, stronger bones and improved physical condition; and, on a psychological level, improved cognition, better school performance, increased cognitive function and improved mental health, which generates psychological wellbeing.

However, there is limited information on the effects of PA on the development and psychological wellbeing of children and adolescents in the context of PE and school sports. It is therefore necessary to identify different strategies and interventions for developing psychological wellbeing in the literature. The primary objective of this paper is to explore studies that seek to promote psychological wellbeing among schoolchildren through PE and school sports and to identify conceptualisations of psychological wellbeing in this specific context. 

This review has two objectives. Firstly, it seeks to address the following questions.

What are the characteristics of studies on psychological wellbeing interventions in PE and school sports?

What are their objectives? 

What does the literature report on the outcomes of interventions aiming to improve psychological wellbeing?

Secondly, this review attempts to analyse how psychological wellbeing is conceptualised in this context and to provide a definition of the concept based on the findings.

## 2. Materials and Methods 

### 2.1. Search Strategy

This review was carried out following the protocol outlined in the PRISMA statement. A comprehensive search was conducted in four databases: WOS, SPORTDiscus (EBSCO), SCOPUS and ERIC (Proquest). A number of articles were selected considering bibliography of reference research (6). Individual searches of all studies published up to September 2019 were performed in each database following the PICO protocol as used by Opstoel et al. [9] (P = Population, I = Intervention, C = Comparison, O = Outcomes).

- P = child, children, boys, girls, adolescents
- I = physical education
- C = no comparison group was added to the search
- O = psychological wellbeing, eudaimonic wellbeing.

The search terms used were “psychological wellbeing” and “eudaimonic wellbeing”, in combination with “AND” and the search terms “physical education” and “child”, “children”, “boys”, “girls”, and “adolescents”. Searches were conducted in English and Spanish. Only original articles were included in the study.

### 2.2. Selection Criteria

Potentially relevant studies for this review were checked against the following selection criteria: (a) the study had been published in an international peer-reviewed journal; (b) the study covered interventions with children and adolescents aged between 6 and 18 years old; (c) the study explored the relationship between PE or school sports and psychological wellbeing; and (d) a full-text version was available in English and/or Spanish.

Regarding the first criterion, interventions implemented in the school setting (PE classes and in-school and extracurricular sports activities) were eligible. Regarding the second criterion, interventions with children and adolescents at all stages of formal schooling within the aforementioned age range were also considered for inclusion. In the event that the studies included individuals outside that age range, only articles with the majority of participants within that age range were eligible. 

Articles were excluded following Opstoel’s criteria [9]:

- Studies involving a specific population with any type of physical, cognitive or psychological impairment.
- Articles not providing primary data (non-interventions), as they do not ensure methodological and statistical rigour (reviews, conceptual articles, conference proceedings, editorials, doctoral theses, books, opinion articles, etc.).
- Instrument validations.

**Figure 1:**
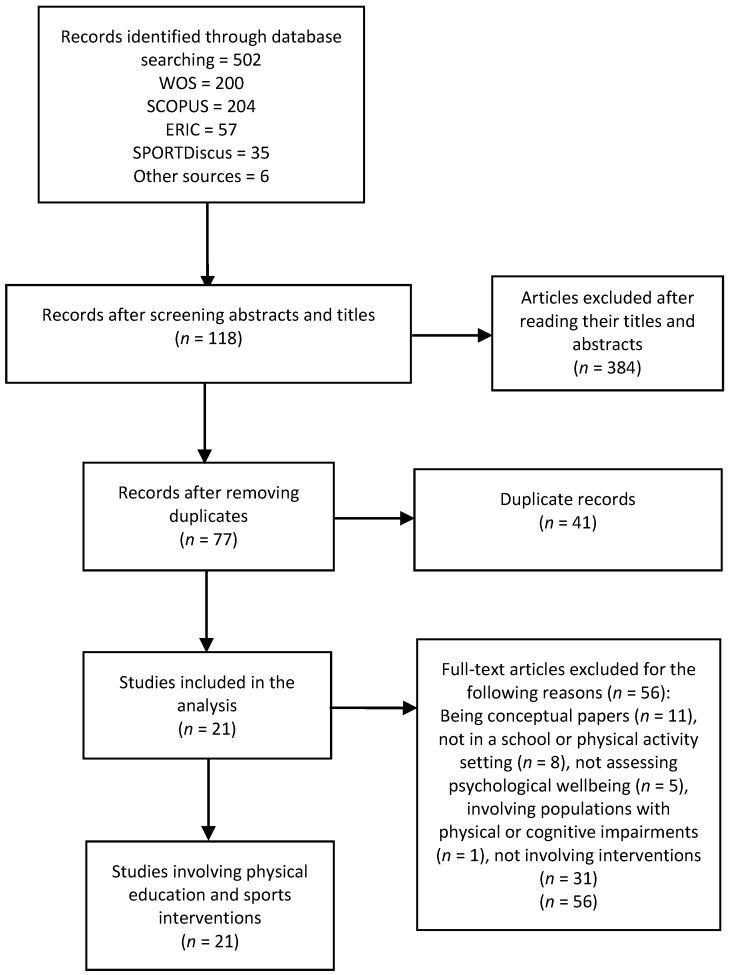
Flow chart of the sampling process.

Duplicates were discarded. The study selection process consisted of screening the titles and abstracts identified during the search. Potentially relevant full-text studies were independently checked for eligibility by two authors, J.P.-C. and R.P.-O. Discrepancies in the selection of the articles were discusses until a consensus was reached. Figure 1 shows the sampling process used. After removing duplicates and excluding records by abstract and title, a total of 21 articles were retrieved.

### 2.3. Data Extraction and Reliability

Pilot test forms were used to extract data from the studies. A content analysis of the articles included in this review was also performed. Subsequently, the data were discussed and confirmed by the researchers. The following categories were defined: authors, year, journal (volume and issue), country, objectives, sample size, characteristics of the participants, duration of the study, instruments used to assess psychological wellbeing, and results (Table 1).

The criteria for assessing the quality of the studies included were adapted from the Consolidated Standards of Reporting Trials (CONSORT) Statement [41] as used by Pozo et al. [42]. The quality assessment criteria were: (a) description of the programme, (b) number of participants, (c) inclusion of the journal of publication in the Journal Citation Reports, (d) duration of the programme, (e) description of the methodology; (f) definition of psychological wellbeing.

Each item was rated from 0 to 2 based on the criteria outlined in Figure 1. A total score was calculated for each study depending on the number of positive items it contained. Studies with a total score of 9 or higher were considered to be of high quality (HQ); studies with a total score of 5–8 were considered to be of average quality (AQ); studies with a total score lower than 4 were considered to be of low quality (LQ). Details are shown in Table 2. 

The risk of bias is difficult to ascertain in qualitative, social science studies. Version 5.1.0 of the Cochrane handbook emphasises that, in many situations, it is not practical or possible to blind participants or study staff in the intervention group.

## 3. Results

### 3.1. Sample

The total number of participants in the studies reviewed was 10,357, ranging from 23 [40] to 3124 [38].

The ages of participants ranged from 7 to 18 years old. Two interventions involved children under 10 [22,36], eight interventions involved children aged 10–15 years old, eleven interventions involved children around 15 years old, one intervention involved children aged 11–16 years old [21], one intervention involved children aged 12–15 years old [33], one intervention involved children aged 12–18 years old [25], and one intervention involved children aged 13–16 years old [29].

Regarding participants’ levels of education, 5 of the 21 studies focused on primary education students, 15 studies focused on secondary education students, and 1 study focused on primary and secondary education. 

### 3.2. Countries

Most of the studies were conducted in the United Kingdom (4/21) and the United States (4/21), followed by Australia (2/21), China (2/21), Turkey (2/21), Canada (1/21), Denmark (1/21), Greece (1/21), Finland (1/21), South Africa (1/21), Spain (1/21), and Sweden (1/21).

### 3.3. Duration of the Studies

The duration of the interventions ranged from 3 days [37] to 36 weeks [38]. Within that range, four of the 21 studies lasted 8 weeks, three lasted 10 weeks, two lasted 6 weeks, one lasted one week, one lasted 12 weeks, one lasted 13 weeks, one lasted 14 weeks, one lasted 18 weeks, one lasted 20 weeks, one lasted 23 weeks, and one lasted 24 weeks. One study [28] indicated that the pilot study, intervention, evaluation and follow-up lasted for 2 years.

The number of sessions ranged from 4 [36] to 35 [34]. It is important to note that 13 of the 21 studies provided information on the number of sessions conducted in their interventions/programmes.

### 3.4. Instruments Used to Assess Wellbeing

A variety of instruments were used to assess wellbeing depending on how wellbeing was conceptualised. Only 16 of the 21 articles mentioned instruments for measuring wellbeing: the KIDSCREEN-10, -27, and -52 measures (4/16); the Flourishing Scale (3/16); the Positive Affect and Negative Affect Schedule for Children (3/16); the Warwick-Edinburgh Mental Wellbeing Scale (2/16); Ryff’s Psychological Wellbeing Scale (2/16); the SF-12v2 (1/16); the Profile of Mood States (1/16); the Perceived Stress Scale (1/16); the Inventory of Positive Psychological Attitudes (1/16); Harter’s Self-Perception Profile for Children (1/16); the Danish national survey of wellbeing in the school-aged population (1/16); the Personally Expressive Activities Questionnaire (1/16); the British Panel Household Survey (BHPS-Y) (1/16); the Perceived Behavioral Control Questionnaire (1/16); the Self-Efficacy Questionnaire (1/16); the Physical Self-Description Questionnaire (1/16). 

### 3.5. Conceptualisation of Psychological Wellbeing

A variety of conceptualisations of psychological wellbeing were presented in the studies. They were so diverse that there was no consensus among the 21 articles reviewed on the definition of psychological wellbeing in the context of PE and school sports. Some definitions focused on self-confidence, improvements in mood (feeling happier or less sad), self-discipline and goal-setting [21], while other definitions revolved around a broader conceptualisation of wellbeing from the hedonic or eudaimonic perspective [32,39]; as well as health-related quality of life [31], specifically mental health [30]; self-concept and mental health (depression and anxiety) [34]; psychosocial wellbeing: mood states, affects, and perceived stress [35]; self-esteem, intrinsic motivation and attitudes towards dance and group PA [23]; positive feelings towards five domains in life: school, work, family, appearance and friends [29]; flourishing, establishing relationships, self-esteem, purpose in life and optimism [24,27,32] health-related quality of life, positive and negative affects, emotional intelligence and social anxiety [33]; positive thoughts and emotions [30]; self-acceptance and human fulfilment [25]; individuals’ awareness of their own abilities to overcome stress in life, be productive, and contribute their skills to the community [20]; development of human potential and self-realization, which encompasses developing self-acceptance, positive relations with others, self-determination, environmental mastery, purpose in life, and personal growth [26]. Five of the studies analysed did not provide a clear definition of the concept of psychological wellbeing [22,28,36,38,40].

### 3.6. Objectives of the Studies 

The objectives most frequently addressed in the articles related to assessing the effects of the programmes on participants (12/21), specifically: the effectiveness of a positive youth development-based sports mentorship programme on wellbeing [30]; the effects of PA and avoiding screen time on wellbeing [32]; the effects of moderate-to-vigorous physical activity (MVPA) on the wellbeing of PE students [27]; the effect of a health education programme on participants’ perceptions of their quality of life [31]; the effectiveness of a randomised, controlled intervention on wellbeing [39]; the effect of a hip-hop dance programme on adolescent wellbeing [21]; the effects of a pedometer-based physical activity intervention on the psychological wellbeing of overweight adolescents [25]; the effects of a health club approach on adolescents [34]; the effect of sports education on the psychological wellbeing of high school students [26]; the effect of a curriculum-based physical activity intervention on primary school students [22], and the effects of running on wellbeing-related variables [36]. Another study sought to evaluate the effect of sports on wellbeing in general [20], while two studies aimed to develop, implement, and evaluate physical activity interventions to improve psychosocial wellbeing [38] and reduce sedentary behaviour [28]. Two other studies sought to assess the effectiveness of PA and sports induction protocols and programmes on psychological wellbeing [24,37]. Three studies aimed to assess the impact of specific programmes on variables related to wellbeing and PA [23,29,33] while another study sought to explore the implementation and short-term outcomes of a responsibility-based physical activity programme that was integrated into an intact high school PE class [40]. Finally, one study aimed to assess whether integrating yoga into the secondary school curriculum had a preventive effect on wellbeing among secondary school students [35].

### 3.7. Results of the Studies

The articles reviewed mainly reported on the effects of the programmes studied. In three of them [20,24,36], no statistically significant differences in wellbeing were found post-intervention. In five of them [21,27,28,39,40], the authors proposed their respective intervention programmes as strategies for promoting PA and psychosocial variables; however, they failed to provide any results on wellbeing per se. Additionally, Ho et al. [30], McNamee et al. [34], and Connolly et al. [23] described the effects of their programmes on mental health, wellbeing, and other psychological, physical, and PA-related variables among adolescents. In the same vein, Bakır & Kangalgil [20] stated that although no changes in participants’ positivity were identified, there were changes in the mental wellbeing of participants who took part in sporting activities, which was also assessed by Smedegaard et al. [38]. Karasimopoulou et al. [31] reported that children in the experimental group significantly improved their perceptions of physical wellbeing, family life, financial aspects, friends, school life and social acceptance, with better perceptions of autonomy than the control group in the final measurement. In turn, Lubans et al. [32] and Slee & Allan [37] linked their results to the fulfilment of basic psychological needs. While Lubans et al. [32] argued that in order to achieve psychological wellbeing, it is necessary to address autonomy, Slee & Allan [37] argued that psychological wellbeing could be related to self-determination. Other results were linked to the effect of the programmes on academic performance, wellbeing and brain development [22]; improved physical condition, satisfaction with appearance, more positive attitudes towards school and friends, and greater environmental awareness [29]. Finally, Noggle et al. [35] reported that although PE-as-usual students showed decreases in primary outcomes, yoga students maintained or improved them, echoing the findings of Luna et al. [33] regarding subjective wellbeing and emotional intelligence, and Gül et al. [26], who reported that PA and sports had an effect on the individual development of the different dimensions of psychological wellbeing.

## 4. Discussion

The aim of this review was to analyse the characteristics, objectives, and results of studies seeking to promote psychological wellbeing among schoolchildren through PE and school sports, as well as to identify different conceptualisations of the construct in this specific context and provide a definition of it.

With regard to the first objective, most of the interventions identified were held at secondary schools within school hours, both in PE classes and during in-school and extracurricular sports activities. This was consistent with results from other studies on programmes targeting this population [10,43]. In addition, the durations of the programmes reviewed were similar to those of other programmes involving children and, especially, adolescents. A systematic review by Opstoel et al. [9] notes that studies on this type of population tend to last between 8 and 28 weeks. However, Rodríguez-Ayllon et al. [44] report that interventions can last from 10 days to 2 years. The studies analysed had multiple, varied objectives that can be grouped into four major categories: (i) to evaluate the effects of the interventions and/or programmes on participants, (ii) to explore correlations between the programmes and wellbeing, (iii) to identify relationships between different variables, and (iv) to explore wellbeing and empirical strategies for programme evaluation. These objectives are shared by other studies on variables linked to wellbeing across different populations and settings, such as: individuals with diabetes and the effectiveness of programmes on wellbeing [45]; pre-schoolers, infants, and adolescents, and the effect of PA on mental health [44]; and the effect of PA on happiness [46]. 

The results obtained from the interventions are linked to other systematic reviews on personal and social growth aspects of PE that seek to explore the effects of PA on psychological wellbeing [43] and improve the psychological and social skills of children and young people to better prepare them for the future [9]. A number of studies have also argued that wellbeing is related to fulfilling basic psychological needs, such as Menéndez-Santurio & Fernández-Río [47], who identified a relationship between social responsibility, basic psychological needs and motivation, and described how these can predict positive relations with others, especially friends. Similar results are reported by Molina, Gutiérrez, Segovia & Hopper [48], who identified a relationship between the implementation of a sports programme and improved basic psychological needs, social relationships and responsibility. In addition, Menéndez-Santurio, Fernández-Río, Cecchini & González-Villora [49] confirm that students who have low wellbeing rates due to victimisation and bullying at school have low levels of satisfaction of basic psychological needs, which supports the relationship between wellbeing, basic psychological needs and self-determination put forward by various authors [12,50,51]. It is important to note that the majority of the studies reviewed used multiple forms of physical activity, such as dance, active play and modified sports, and do not use conventional sports to promote wellbeing. This is in consonance with a review by Sánchez-Alcaraz et al. [52], which discusses the importance for psychosocial development of creating a balance between conventional or more popular sports and other less popular sports and physical activities or exercises offering new experiences for children and adolescents.

There is also a lack of consensus on the definition of psychological wellbeing in the context of PE and school sports. Nevertheless, it is fairly safe to say that more than half of the studies (13 out of 21) linked the construct to Diener’s definition [53], which is related to the concept of hedonism or subjective wellbeing. As a result, these studies assessed variables such as life satisfaction, affects and depression, rather than the dimensions included in Ryff’s IMPD regarding eudaimonic or psychological wellbeing [16,54]. This trend could be explained by the interest in understanding and promoting individual happiness that emerged in the 1980s [14], which was reinforced by Huta & Waterman [55], who pointed out asymmetries and preferences in research on these concepts. Additionally, Cabieses, Obach & Molina [56] argue that producing knowledge from the perspective of subjective wellbeing, that is, looking into life satisfaction and happiness, could be useful when planning public policies for this population. Since subjective wellbeing is associated with immediacy, a large number of studies use this construct. Romero, García-Mas & Brustad [12] point out that psychological wellbeing in the field of PA and sports has not been approached consistently, which may explain the scarcity of studies on this topic. In line with Huta & Waterman [55], we believe that it is necessary to find a compromise definition to inform future research on this topic, as the use of a wide range of definitions produces a wide range of results when analysing and comparing studies focusing on the same concept. 

To this end, we propose the following definition based on the aforementioned findings, Ryff’s theoretical approach to psychological wellbeing [14,16,54,57], and aspects inherent to PA and sports, such as movement and corporeality: “Psychological wellbeing in PA (PWBPA) is the state of optimal psychological functioning in the context of physical activity, which encompasses accepting one’s strengths and limitations, being independent in decision-making and self-assessment, choosing or creating favourable environments, interacting positively with others in PA and sports, developing one’s potential to the fullest, and seeking meaning and purpose in life based on PA values.”

With regard to the limitations of our study, articles in both Spanish and English were included; however, their results only provided information from English-speaking countries, limiting perceptions of the phenomenon to a particular culture, which may have influenced the researchers’ conceptualisation of the phenomenon in this particular population. In addition, the search was limited to interventions involving populations without pre-existing physical or cognitive issues, excluding other articles which may have been relevant to the topic. In light of the limited number of studies on psychological wellbeing in the field of PA, specifically in PE and school sports, it would be helpful to conduct a meta-analysis to identify the effects of PA and sports programmes/interventions on the psychological wellbeing of children and youths in the school setting. It would also be of interest for future research to analyse and review articles on PE and sports from the point of view of different countries and cultures. Also, to deepen knowledge it would be interesting in future research to consider additional threads related to other contexts besides PE classes or sport school, such as different kinds of sports practices and socioeconomic differences between schools. Finally, in order to broaden the field of knowledge, further studies could be carried out to provide information on parents, guardians and agents of socialisation, who are very important in the development of children and adolescents.

## 5. Conclusions

Psychological wellbeing in PE and school sports is a developing field that has drawn increasing attention in recent years, which may be due to the need for PA to improve mental health and quality of life for children and adolescents.

We found that most programmes/interventions involve adolescents, especially in secondary schools. The programmes usually last between 3 days and 36 weeks (9 months or an academic year), and it is English-speaking countries, such as the United States and the United Kingdom, that have conducted the majority of the studies on this topic. There is no consensus as to definitions of the concept, study objectives, methods or tools for assessing psychological wellbeing. As for whether or not PA promotes psychological wellbeing in PE and school sports, the disparate results of the studies analysed do not allow us to draw conclusions. However, there appears to be a relationship between PA, wellbeing and other variables, such as basic psychological needs and quality of life.

From an educational perspective, the authors suggest that future interventions should employ a single definition of psychological wellbeing in PA, such as the one proposed in this paper. This would promote self-realisation and personal growth in children and adolescents by focusing on transcendence rather than on a narrow search for subjective wellbeing, while providing researchers with a common criterion for applying the concept in the context of PA. 

## Figures and Tables

**Table 1:** General overview of the articles included.

Study	Year	Journal (Volume and Issue)	Country	Objectives	Characteristics of the Population	Sample Size	Duration (Weeks)	Instruments Used to Assess Wellbeing	Results
Bakır & Kangalgil [20]	2017	*Journal of Education and Training Studies*, 5(11)	Turkey	To explore the effectiveness of sports on mental wellbeing and positivity.	10th grade high school students	60	20	Psychological wellbeing: Ryff’s Psychological Wellbeing Scale	Based on the study results, significant differences between pre-test and post-test positivity were identified. There were significant differences in mental wellbeing between the sports activities group and the control group.
Beaulac et al. [21]	2011	*Journal of Youth Studies*, 14(8)	Canada	To explore whether a hip-hop dance programme was associated with improved wellbeing for adolescents living in a multicultural, socio-economically disadvantaged urban community in Ottawa.	Youths between 11 and 16 years of age living in a multicultural, socio-economically disadvantaged urban community	63	13	Eudaimonic wellbeing: The Personally Expressive Activities Questionnaire; Hedonic wellbeing: The Positive Affect and Negative Affect Schedule for Children	The findings suggested that the community-based intervention was a promising programme for the promotion of youth psychological, social, and physical wellbeing.
Bunketorp et al. [22]	2015	*Journal of School Health*, 85(10)	Sweden	To explore whether a curriculum-based physical activity intervention correlates positively with children’s academic achievement, psychological well-being, health-related quality of life (HRQoL), fitness, and structural development of the brain.	Primary school students	428	Not reported	Wellbeing: The British Panel Household Survey (BPHS-Y) assesses 5 domains: school, work, family, appearance, and friends at school.	Girls attending the intervention school were more likely to pass national tests in Swedish and Mathematics. The fourth to sixth graders in the intervention school reported lower levels of conduct problems, and the girls were also less likely to report hyperactivity. Girls reported higher levels of emotional problems than boys. Boys in the intervention group had significantly higher levels of estimated maximal oxygen uptake than controls. No difference in hippocampal structure was seen.
Connolly et al. [23]	2011	*Research in Dance Education*, 12(1)	United Kingdom	To assess the physiological and psychological impact of contemporary dance classes on adolescent females.	Females aged 14 recruited from secondary schools	55	Not reported	Health-related quality of life: KIDSCREEN-27; 5 dimensions: physical wellbeing, psychological wellbeing, autonomy & parents, peers & social support, school environment.	There was a statistical increase in areas of physical fitness. Aerobic capacity and upper body strength increased significantly. No significant change occurred in flexibility. Self Esteem statistically increased. No change occurred in Intrinsic Motivation, however motivation scores were higher than average at pre-test stage. Attitudes towards dance were very positive both pre- and post-intervention. This study showed that dance can statistically improve components of physical fitness, and psychological well-being.
Costigan et al. [24]	2016	*Medicine and Science in Sports and Exercise*, 8	Australia	To evaluate the efficacy of two high-intensity interval training (HIIT) protocols for improving cognitive and mental health outcomes (executive function, psychological wellbeing, psychological distress, and physical self-concept) in adolescents.	Secondary school students aged 14–16 years old.	65	24	Does not mention instruments for assessing wellbeing	While results were not significant, small improvements in executive function and psychological wellbeing were evident in the aerobic exercise programme (AEP) group; and moderate improvements in executive function, and small improvements in wellbeing and perceived appearance were observed for the resistance and aerobic programme group. Mean feeling state scores improved from preworkout to postworkout in both HIIT conditions, with significant results for the AEP.
Grace et al. [25]	2015	*African Journal for Physical, Health Education, Recreation and Dance*, 21(4)	South Africa	To determine the effects of a pedometer-based physical activity intervention on the psychological wellbeing and selected physical and physiological indices in overweight/obese adolescents.	Overweight/obese adolescents aged 12-18 years from two secondary schools.	31	Not reported	Mental wellbeing: Warwick-Edinburgh Mental Wellbeing Scale	Our results indicate that a relatively small change in activity caused a statistically significant improvement in cardio respiratory fitness with no significant impact on psychological wellbeing.
Gül et al. [26]	2017	*Journal of Education and Training Studies*, 5(5)	Turkey	To examine the effect of sports education on psychological wellbeing among high school students at the individual, environmental, and self-determination levels.	Secondary school students aged 14–18 years old.	187	16	Does not mention instruments for assessing wellbeing	Regarding the individual development dimensions of the psychological wellbeing scale, there were statistically significant differences between the experimental groups and the control groups, especially post-test.
Ha et al. [27]	2017	*BMC Public Health*, 18(11)	China	To examine the effects of the SELF-FIT intervention on students’ moderate-to-vigorous physical activity (MVPA) MVPA during school physical education.	Secondary school students with an average age of 14 years.	773	Not reported	Does not mention instruments for assessing wellbeing	The SELF-FIT intervention has been designed to improve students’ health and wellbeing by using high-intensity activities in classes delivered by teachers who have been trained to be autonomy-needs supportive. If successful, scalable interventions based on SELF-FIT could be applied in physical education at large.
Hankonen et al. [28]	2016	*BMC Public Health*, 16(1)	Finland	To describe the study protocol for a cluster RCT designed to evaluate the effectiveness, cost-effectiveness, and processes of the Let’s Move It programme compared with the usual curriculum among vocational school students.	Students between the ages of 15 and 17 years from 6 vocational schools.	1123	30	Psychological wellbeing: The Flourishing Scale	Does not report on results.
Hignett et al. [29]	2018	*Journal of Adventure Education and Outdoor Learning*, 18(1)	United Kingdom	To explore the impact of a 12-week surfing programme on a group of children/young people excluded, or at risk of exclusion, from mainstream schooling in the UK.	Young people between the ages of 12 and 16 years, who have either been excluded from mainstream schooling and therefore attend Short Stay Schools, or attend mainstream schools, but are considered at risk of exclusion.	58	12	Does not mention instruments for assessing wellbeing	Results found significant drops in heart rate (suggesting improved fitness), increased satisfaction with appearance, more positive attitudes towards school and friendships, greater environmental awareness and more positive teacher evaluations, post-intervention. A lack of findings in other domains suggests these results were not due to participants simply conforming to demand characteristics.
Ho et al. [30]	2017	*Pediatrics*, 140(4)	China	To assess the effectiveness of a positive youth development (PYD)-based sports mentorship programme on the physical and mental wellbeing of adolescents recruited in a community setting.	Students from 12 secondary schools	664	Not reported	Perceived self-control in physical activity: The Perceived Behavioral Control Questionnaire; Self-efficacy: The Self-Efficacy Questionnaire; Self-concept: The Physical Self-Description Questionnaire	The PYD-based sports mentorship intervention improved healthy adolescents’ mental wellbeing, psychological assets, physical fitness, and physical activity levels.
Karasimopoulou et al. [31]	2012	*Health Education Research*, 27(5)	Greece	To examine the effect of the Health Education Programme ‘Skills for primary school children’ on children’s perceptions about certain dimensions of their quality of life: physical wellbeing, mental wellbeing, moods and emotions, self-concept, leisure–autonomy, family life, financial resources, friends, school environment and social acceptance (bullying).	Students aged 10–12 years from 12 primary schools.	286	23	Does not mention instruments for assessing wellbeing	Children in the experimental group significantly improved their perceptions of physical wellbeing, family life, financial aspects, friends, school life, and social acceptance. On the other hand, children in the control group significantly improved their perceptions for physical wellbeing, whereas they deteriorated them significantly for family life, mood and feelings and social acceptance. Also, children as a whole improved their self-concept. Furthermore, analysis of covariance showed that the experimental group had better perceptions of autonomy than the control group in the final measurement.
Lubans et al. [32]	2016	*Journal of Adolescent Health*, 58(2)	Australia	To explore the effect of the ‘Active Teen Leaders Avoiding Screen-time’ (ATLAS) intervention on psychological wellbeing in adolescent boys and to examine the potential mediating mechanisms that might explain this effect.	Adolescent boys with a mean age of 12.7 years from schools located in low-income communities.	361	Not reported	Positive thoughts and emotions: The Warwick-Edinburgh Mental Wellbeing Scale	The intervention effect on wellbeing was small but statistically significant. Within a multiple mediator model, changes in autonomy needs satisfaction, recreational screen-time and muscular fitness significantly mediated the effect of the intervention on psychological wellbeing.
Luna et al. [33]	2019	*International Journal of Environmental Research and Public Health*, 16(1821)	Spain	To evaluate the impact of a physical-sport education pilot programme on adolescents’ subjective wellbeing (health-related quality of life, positive affect and negative affect), trait emotional intelligence and social anxiety.	Compulsory secondary education students aged 12–15 years	113	16	Psychosocial wellbeing: Harter’s Self-Perception Profile for Children; General wellbeing and wellbeing at school: 13 questions from the Danish national survey of wellbeing in the school-aged population; Health-related quality of life: KIDSCREEN-27	The physical-sport education pilot programme promoted significant improvements in a specific indicator of subjective wellbeing and trait emotional intelligence in the experimental group.
McNamee et al. [34]	2016	*European Physical Education Review*, 23(4)	USA	To demonstrate the efficacy and feasibility of an innovative physical education programme, referred to as a health club (HC) approach, in a high school setting.	Adolescent girls in a high school setting, aged 14–15 years.	1970	35	Psychosocial wellbeing: Mood states: The Profile of Mood States-Short Form; Affects: The Positive Affect and Negative Affect Schedule for Children; Perceived stress: The Perceived Stress Scale; Positive psychology: The Inventory of Positive Psychological Attitudes-32R.	The HC approach produced high levels of moderate-to-vigorous physical activity (MVPA). Significant differences were found in participants’ flexibility and cardiovascular fitness at the conclusion of the 14-week programme. Participants reported greater perceived control over their PA, improvements in their physical self-concept across several domains, more positive feelings about appearance and strength and more positive global statements about their physical selves at post-test.
Noggle et al. [35]	2012	*Journal of Developmental & Behavioral Pediatrics*, 33(2)	USA	To test feasibility of yoga within a high school curriculum and evaluate preventive efficacy for psychosocial wellbeing.	Grade 11 or 12 students with a mean age of 17 years.	51	28	KIDSCREEN-52	Although PE-as-usual students showed decreases in primary outcomes, yoga students maintained or improved.
Sifers & Shea [36]	2013	*Journal of Clinical Sport Psychology*, 7	USA	To measure broader emotional and behavioural functioning along with the self-esteem and body image of the participants in the GOTR/T programme at the beginning and upon completion of the programme to further evaluate the programme’s effect on self-esteem, body image, and emotional and behavioural functioning.	Girls, ages 8 to 13	111	4	Psychological wellbeing: The Flourishing Scale	Results suggest GOTR/T may help improve self-esteem in relation to physical appearance and body image. Improvements in other domains were not found.
Slee & Allan [37]	2019	*Sports*, 7(134)	United Kingdom	To investigate the efficacy of three contrasting induction programmes for facilitating improvements in children’s psychological wellbeing and self-determination during their transition into secondary school.	Primary school children aged 11.	100	Not reported	Psychological wellbeing: Ryff’s Psychological Wellbeing Questionnaire	A bespoke outdoor adventure (OA) residential programme achieved the strongest scale of change in children’s psychological wellbeing and self-determination compared to a generic OA residential and a non-OA school-based induction programme.
Smedegaard et al. [38]	2016	*BMC Public Health*, 16(1127)	Denmark	To develop, implement, and evaluate a multi-component, school-based, physical activity intervention to improve psychosocial wellbeing among school-aged children and youths.	Children and youths from the 4th to the 6th grade (10–13 years).	3124	Not reported	Psychological wellbeing: The Flourishing Scale	The intervention focuses on the mental benefits of physical activity at school, which has been a rather neglected theme in health promotion research during recent decades.
Standage et al. [39]	2013	*BMC Public Health*, 13(666)	United Kingdom	To determine the effectiveness of the BtBYCB programme on (i) pupils’ wellbeing, self-perceptions, self-esteem, aspirations and learning strategies; and (ii) changes in modifiable health-risk behaviours (i.e., physical activity, diet, smoking and alcohol consumption).	Primary school children aged 11–13.	711	13	Physical and mental wellbeing: the Chinese version of the SF-12v2.	The findings of this work provide insight into the effectiveness of an innovative and child-centered programme. The research informs improvements to the BtBYCB programme as well as other interventions targeting child/youth health and wellness.
Wright & Burton [40]	2008	*Journal of Teaching in Physical Education*, 27(2)	USA	To systematically explore the implementation and short-term outcomes of a responsibility-based physical activity programme that was integrated into an intact high school PE class.	African American students in an urban high school.	23	20	Subjective wellbeing: KIDSCREEN-10; Affects: The Positive Affect and Negative Affect Schedule for Children	Five themes characterised the programme: (a) establishing a relevant curriculum, (b) navigating barriers, (c) practising life skills, (d) seeing the potential for transfer, and (e) creating a valued programme.

**Table 2:** Quality of the studies.

Study	Description of the Programme	Number of Participants	Included in JCR	Duration of the Programme	Description of the Methodology	Definition of Psychological Wellbeing	Overall Score	Quality Level
Bakır & Kangalgil [20]	0	0	2	1	0	2	5	AQ
Beaulac et al. [21]	1	0	2	1	1	2	7	AQ
Bunketorp et al. [22]	1	1	2	2	1	0	7	AQ
Connolly et al. [23]	2	0	1	0	2	2	7	AQ
Costigan et al. [24]	2	0	2	0	2	2	8	AQ
Grace et al. [25]	1	0	0	0	1	2	4	LQ
Gül et al. [26]	2	1	2	0	2	2	9	HQ
Ha et al. [27]	2	2	2	0	2	2	10	HQ
Hankonen et al. [28]	2	2	2	0	2	0	8	AQ
Hignett et al. [29]	1	0	1	1	1	2	6	AQ
Ho et al. [30]	2	2	2	2	2	2	12	HQ
Karasimopoulou et al. [31]	2	1	2	2	2	2	11	HQ
Lubans et al. [32]	1	1	2	2	1	2	9	HQ
Luna et al. [33]	2	1	2	0	2	2	9	HQ
McNamee et al. [34]	1	2	2	1	1	2	9	HQ
Noggle et al. [35]	1	0	2	1	1	2	7	AQ
Sifers & Shea [36]	1	1	1	0	1	0	4	LQ
Slee & Allan [37]	2	0	1	0	2	2	7	AQ
Smedegaard et al. [38]	2	2	2	2	2	0	10	HQ
Standage et al. [39]	2	2	2	0	2	2	10	HQ
Wright & Burton [40]	2	0	2	1	2	0	7	AQ

Parameter 1: Does the study provide a detailed description of the implementation? 0: No, 1: Yes, but it is incomplete or inaccurate, 2: Yes. Parameter 2: The number of participants: 0: fewer than 100 participants, 1: between 100 and 500 participants, 2: more than 500 participants. Parameter 3: Is the journal of publication included in the Journal Citation Reports? 0: No, 1: It is included in the Scimago Journal Rank 2: Yes. Parameter 4: Duration of the intervention: 0: less than 3 months, 1: between 3 and 4 months, 2: more than 4 months. Parameter 5: Does the study provide a detailed description of the methodology? 0: No, 1: Yes, but it is incomplete or inaccurate, 2: Yes. Parameter 6: Does the study provide a definition of wellbeing? 0: No, 1: Yes, but the definition is vague, 2: Yes, and the definition is detailed.
